# Staged approach to bilateral severe carotid stenosis: a case report and literature review

**DOI:** 10.3389/fstro.2025.1594351

**Published:** 2025-08-25

**Authors:** Heitor Cabral Frade, Manmeet Kaur, Julia Aigbogun, Muhammad Zeeshan Memon, Arun Chhabra, Akm Muktadir, Hashem Shaltoni

**Affiliations:** Department of Neurology, University of Texas Medical Branch, Galveston, TX, United States

**Keywords:** carotid artery stenosis, angioplasty, stenting, carotid artery disease, bilateral carotid artery stenosis

## Abstract

**Introduction:**

Carotid atherosclerotic disease (CAD) is a major cause of stroke, often requiring a combination of medical and surgical interventions. Current guidelines have established well the role of interventions such as carotid endarterectomy and carotid artery stenting (CAS) for unilateral carotid disease. However, there is still a paucity of evidence on the timing, procedural order, and complication rate of these procedures when there is bilateral carotid involvement. Hyperperfusion syndrome (HPS), with or without associated intracerebral hemorrhage, although rare, is a major source of morbidity and mortality after carotid interventions, especially in the setting of bilateral CAD. In select cases, staged bilateral CAS (BCAS) appears to attenuate periprocedural risks, including HPS.

**Case report:**

A 62-year-old male presented with acute dysarthria and right-sided face and upper extremity weakness, amounting to an initial National Institutes of Health Stroke Scale (NIHSS) score of 6. Emergent neuroimaging revealed a dense left MCA sign, complete occlusion of the left proximal internal carotid artery (ICA), and severe stenosis of the contralateral ICA. The patient received intravenous thrombolysis and underwent perfusion imaging for possible mechanical thrombectomy. Although the imaging was favorable for endovascular recanalization, the patient continued to clinically improve to an NIHSS score of 3 during angiography, which showed interval recanalization of left proximal ICA, so the procedure was aborted in favor of a delayed staged BCAS. On the day of the first procedure, angiography revealed interval recanalization of the distal ICA and collateral flow to the middle cerebral artery territory associated with early hyperemia. The risks of symptomatic CAS in light of these findings were discussed with the patient, and a shared decision was made to first pursue endovascular treatment of the asymptomatic severe right CAD, followed by treatment of the symptomatic left CAD, to avoid periprocedural complications such as HPS. The patient continued to improve clinically after both procedures and was able to attain functional independence and resume all previous activities following interventions.

**Conclusion:**

This case and literature review suggest that, although both simultaneous and staged BCAS may be feasible treatment options for bilateral CAD, staged BCAS appears to have fewer periprocedural complications such as HPS.

## Introduction

Carotid atherosclerotic disease (CAD) is responsible for 15%−20% of strokes (Chaturvedi et al., 2005), which has motivated several interventional trials over the last decades. Although relatively rare to justify screening in asymptomatic individuals, CAD in its various forms—intima-media thickening, carotid plaque, carotid stenosis—has increased by ~60% between 2000 and 2020 (Song et al., 2020).

Several guidelines have addressed carotid artery stenosis treatment with intensive medical management or surgical management based on symptoms and severity of stenosis (Abbott et al., 2015). In acute symptomatic occlusions, carotid artery stenting (CAS) and carotid endarterectomy (CEA) with or without prior intravenous thrombolysis (IVT) provide the best recanalization and functional outcomes when compared to IVT or intra-arterial thrombolysis alone (Bonati et al., 2021). However, there is a paucity of evidence addressing symptomatic CAD when there is bilateral severe carotid stenosis, which is known to have higher risks of cerebral hyperperfusion syndrome (HPS) with or without intracerebral hemorrhage (ICH; Ouriel et al., 1999; Son et al., 2013).

HPS is a rare but potentially fatal complication of carotid revascularization through different methods. It is attributed to a post-procedural increase in cerebral blood flow associated with impaired cerebrovascular autoregulation. Clinically, it may present with headache, seizures, or transient neurologic dysfunction. Perfusion imaging may reveal a significant interval increase in cerebral blood flow. In some cases, HPS may be associated with ICH and almost always with poor outcomes (Moulakakis et al., 2009).

Multiple strategies for how to approach bilateral carotid stenosis with different combinations of simultaneous or staged CEA and CAS have been reported by different centers (Fukuda et al., 2003; Henry et al., 2005; Liu et al., 2015; Oshita et al., 2020). In a large prospective study of 747 patients with unilateral and bilateral carotid disease, 78 patients were treated for bilateral CAD by initially stenting the symptomatic carotid, followed by stenting the contralateral carotid, and had complication rates similar to the patients with unilateral CAD (Diehm et al., 2008).

Although intervention on the symptomatic carotid artery is still the standard approach in cases of bilateral severe carotid stenosis, there might be a role for an intervention of the asymptomatic carotid artery first in select cases as has been reported in a more recent case series. Eight patients with bilateral carotid stenosis underwent simultaneous CEA and CAS in two distinct orders according to clinical and angiographic characteristics. Patients who had a favorable angiographic profile underwent CEA of the symptomatic side followed by contralateral CAS. If pre-procedure angiography showed blood flow to the symptomatic anterior cerebral artery (ACA) coming from contralateral circulation via the anterior communicating artery (Acomm), the contralateral carotid artery would be intervened first by CAS, followed by CEA of the symptomatic side. Although none of the eight participants suffered ischemic complications or death, one of the five patients who had CEA of the symptomatic side first developed HPS (Xu et al., 2016).

Staged bilateral carotid stenting is a technique used in treating bilateral CAD that involves performing angioplasty and stenting on one carotid artery at a time, with an interval between procedures. This approach has been suggested in cases of bilateral carotid artery stenosis to reduce the risk of complications associated with simultaneously treating both arteries, such as intraoperative hemodynamic depression, HPS, or ICH.

We hereby report a case of acute stroke associated with severe bilateral carotid stenosis that was managed with IVT followed by staged bilateral CAS, starting with the asymptomatic carotid artery.

## Case report

**Figure 1:**
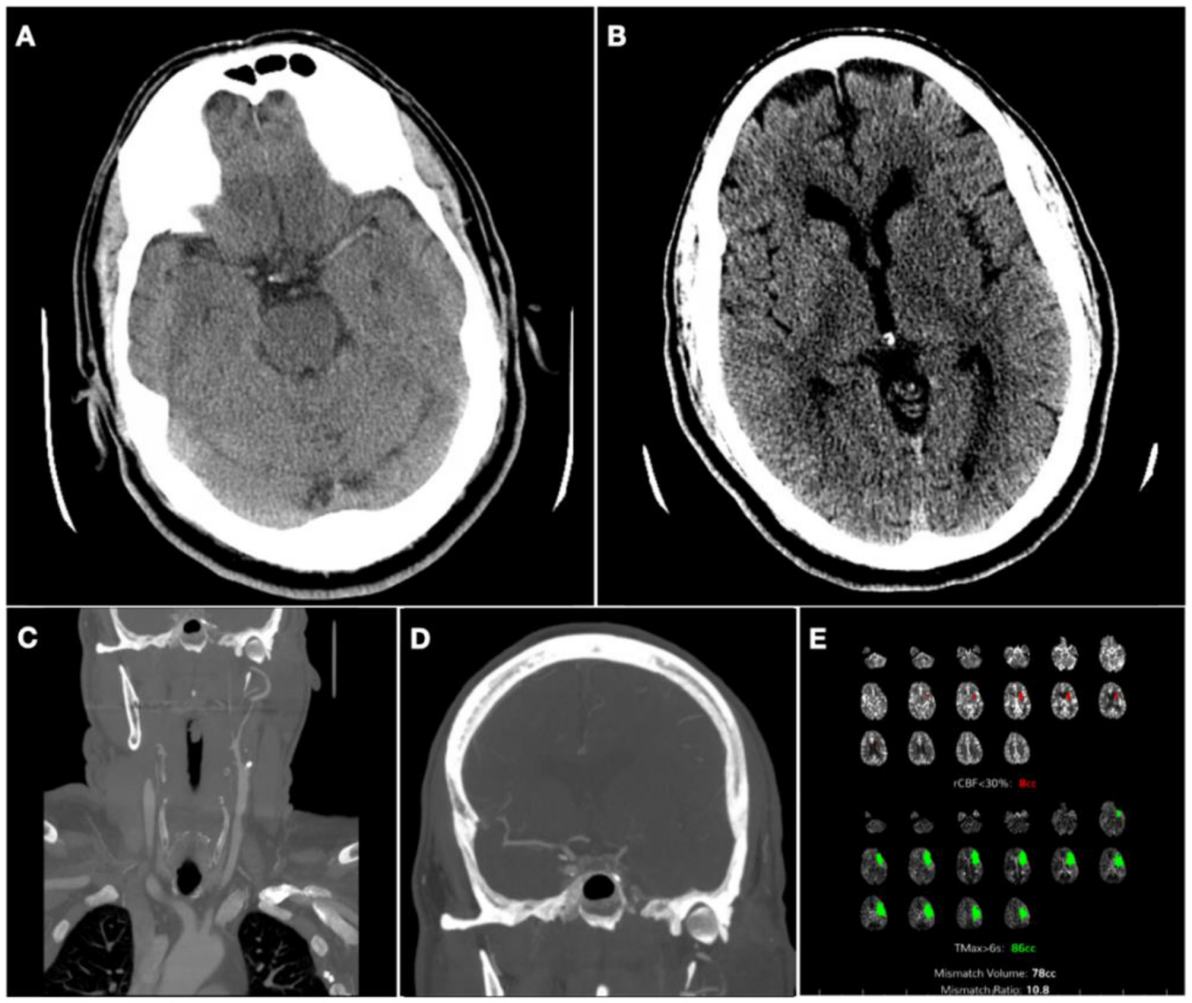
Pre-thrombolytic non-contrast computerized tomography (CT), CT angiogram (CTA), and CT perfusion (CTP). **(A)** Axial CT head showing left dense middle cerebral artery (MCA) sign; **(B)** Axial CT Head, showing decreased gray-white matter differentiation on left lentiform nucleus; **(C)** CTA of neck coronal view: proximal left internal cerebral artery occlusion; **(D)** flow reconstitution of distal ICA flow and occlusion of proximal left MCA. **(E)** CTP showing core of 8cc, penumbra of 86cc, and mismatch ratio 10.8 on the left MCA territory.

A 62-year-old male with a history of hypertension, atrial fibrillation post-ablation not on anticoagulation, and HIV on highly active antiretroviral therapy woke up with dysarthria and right-sided upper extremity and facial weakness. He was immediately taken to our hospital, ~7.5 h after last seen normal and 4 h from the midpoint of sleep. The National Institute of Health Stroke Scale (NIHSS) score at presentation was 6. Non-contrast head computerized tomography showed a dense left middle cerebral artery (MCA) sign (Figure 1A) and decreased gray-white matter differentiation on the left lentiform nucleus, with an Alberta Stroke Program Early CT Score of 9 (Figure 1B). Computed tomography (CT) angiogram showed severe right ICA stenosis and complete occlusion of the left proximal ICA (Figure 1C). There was a tandem occlusion of the M1 segment of the left MCA, but the left proximal ACA was perfused via a patent Acomm (Figure 1D).

CT perfusion (CTP) was pursued to guide acute management, and it showed a core infarct volume of 8cc in the left MCA territory and a penumbra volume of 86cc, with a mismatch volume of 78cc and a ratio of 10.8 (Figure 1E). Upon those findings, the patient received IVT and was taken to the neurointerventional suite for possible mechanical thrombectomy (MT) of the left ICA.

**Figure 2:**
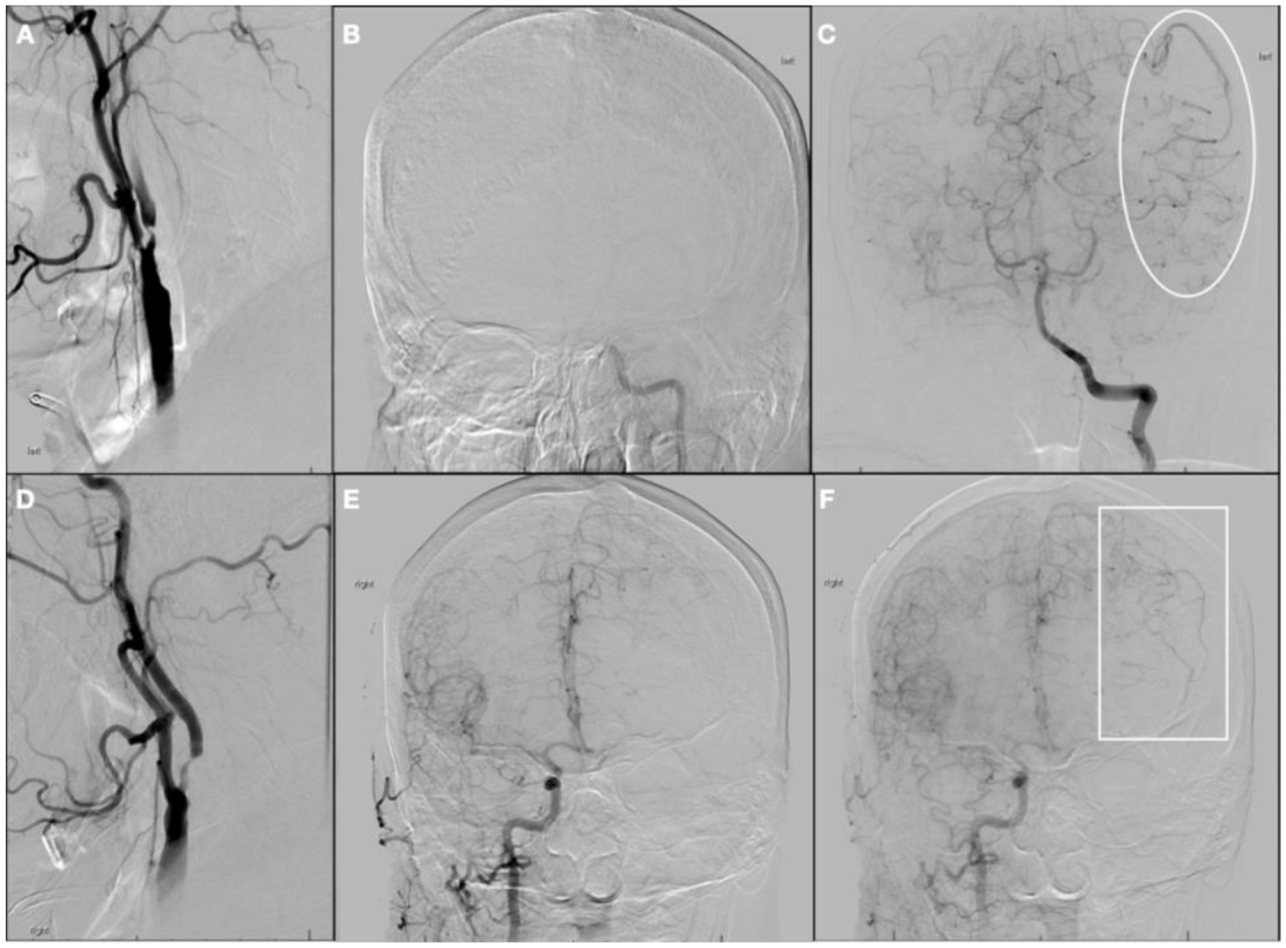
Digital subtraction angiogram at initial presentation. **(A)** Left common carotid artery (CCA) injection, neck lateral view; **(B)** Left CCA injection, head anterior-posterior (AP) view showing non-opacification of terminal left internal carotid artery (ICA); **(C)** Left vertebral artery injection, head AP view, with collateral supply to middle cerebral artery (MCA) territory on late arterial phase (*ellipse*); **(D)** Right CCA injection, neck lateral view; **(E)** Right CCA injection, head AP view showing cross-filling of L anterior cerebral artery on early arterial phase; **(F)** Right CCA injection, head AP view showing collateral supply to MCA territory on late arterial phase (*rectangle*).

During the cerebral angiogram, performed under moderate sedation, he was noted to have progressive improvement of symptoms, with near-total resolution of expressive aphasia and dysarthria and an NIHSS score of 3. Digital subtraction images revealed a near-complete occlusion of the proximal left ICA (Figure 2A), with tandem occlusion of the left terminal ICA (Figure 2B). There was flow to the MCA territory through leptomeningeal collaterals from the posterior cerebral arteries (Figure 2C). There was also severe proximal right ICA stenosis (Figure 2D). There was partial collateral flow to the left terminal carotid branches via the patent anterior communicating complex (Figure 2E) and leptomeningeal collaterals off the left anterior cerebral arteries (Figure 2F). The risks of acute endovascular intervention were deemed to outweigh the benefits in the setting of rapidly improving symptoms and NIHSS score of 3, upon partial recanalization after IVT, so the procedure was aborted.

**Figure 3:**
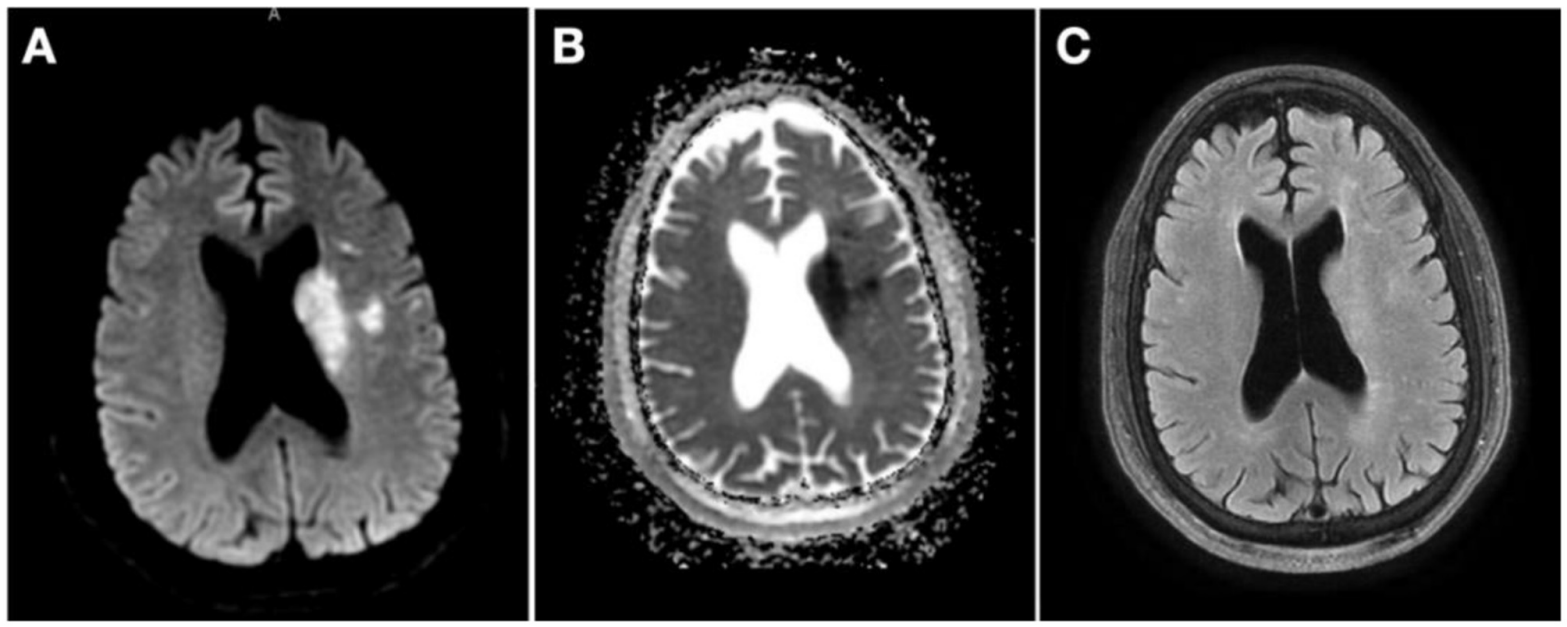
Magnetic resonance imaging (MRI) 12 h from presentation. **(A)** MRI diffusion-weighted imaging showing multiple hyperintense foci on left basal ganglia and left subcortical frontal lobe; **(B)** MRI apparent diffusion coefficient with corresponding hypointensity in the same territories; **(C)** MRI fluid-attenuated inversion recovery showing mild hyperintensity in the same territories.

The patient was admitted for further stroke management and remained clinically stable, with residual deficits of mild right upper extremity weakness and mild expressive aphasia. Brain magnetic resonance imaging (MRI) obtained ~12 h after presentation confirmed small acute infarcts on the left subcortical frontal lobe and basal ganglia (Figures 3A–C). The patient was started on dual antiplatelet therapy with aspirin and clopidogrel 24 h after IVT.

**Figure 4:**
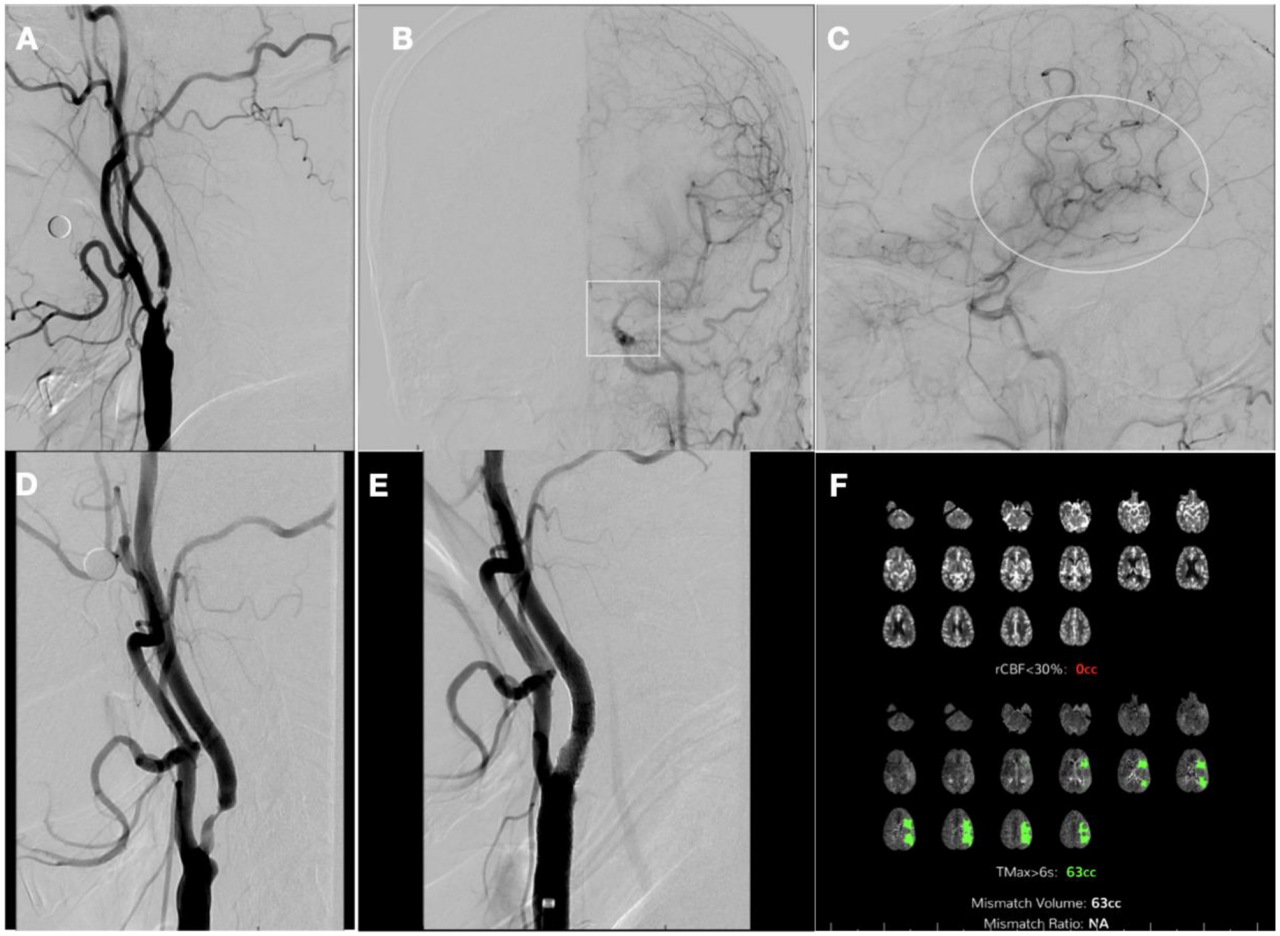
Digital subtraction angiography 1 week after initial presentation and post-stenting computerized tomography perfusion (CTP). **(A)** left common carotid artery (CCA) injection, neck lateral view; **(B)** left CCA injection, head anterior–posterior view showing terminal left internal carotid artery (ICA) recanalization (*square*); **(C)** left CCA injection, lateral view showing early hyperemia on left middle cerebral artery (MCA) territory (*ellipse*); **(D)** right CCA injection, neck lateral view pre-stenting; **(E)** right CCA injection, neck lateral view post-stenting; **(F)** CTP 1 day after right ICA stenting showing no core and penumbra of 63cc on left MCA territory.

One week later, the patient underwent a repeat diagnostic cerebral angiogram to reevaluate severe bilateral carotid stenosis, which revealed persistent severe cervical left ICA stenosis (Figure 4A) but interval recanalization of the left ICA terminus and associated early hyperemia within left MCA territory (Figures 4B, C). The risks of revascularization of the proximal left ICA, given the risk of hyperperfusion, were discussed with the patient, as well as the potential benefit from contralateral ICA stenting in light of the interhemispheric cross-filling. The decision was made to pursue two-stage stenting, starting with the asymptomatic severe right proximal ICA stenosis, followed by the left ICA. The patient underwent successful right ICA stenting without complications (Figures 4D, E) and had an uneventful overnight ICU stay with no neurological or hemodynamic alterations. A repeat CTP the next day showed no core yet a still large penumbra area at risk (Tmax > 6 s) on the MCA territory, 63cc (Figure 4F). He was discharged that day following the procedure with plans to return to undergo stenting of the left internal carotid.

**Figure 5:**
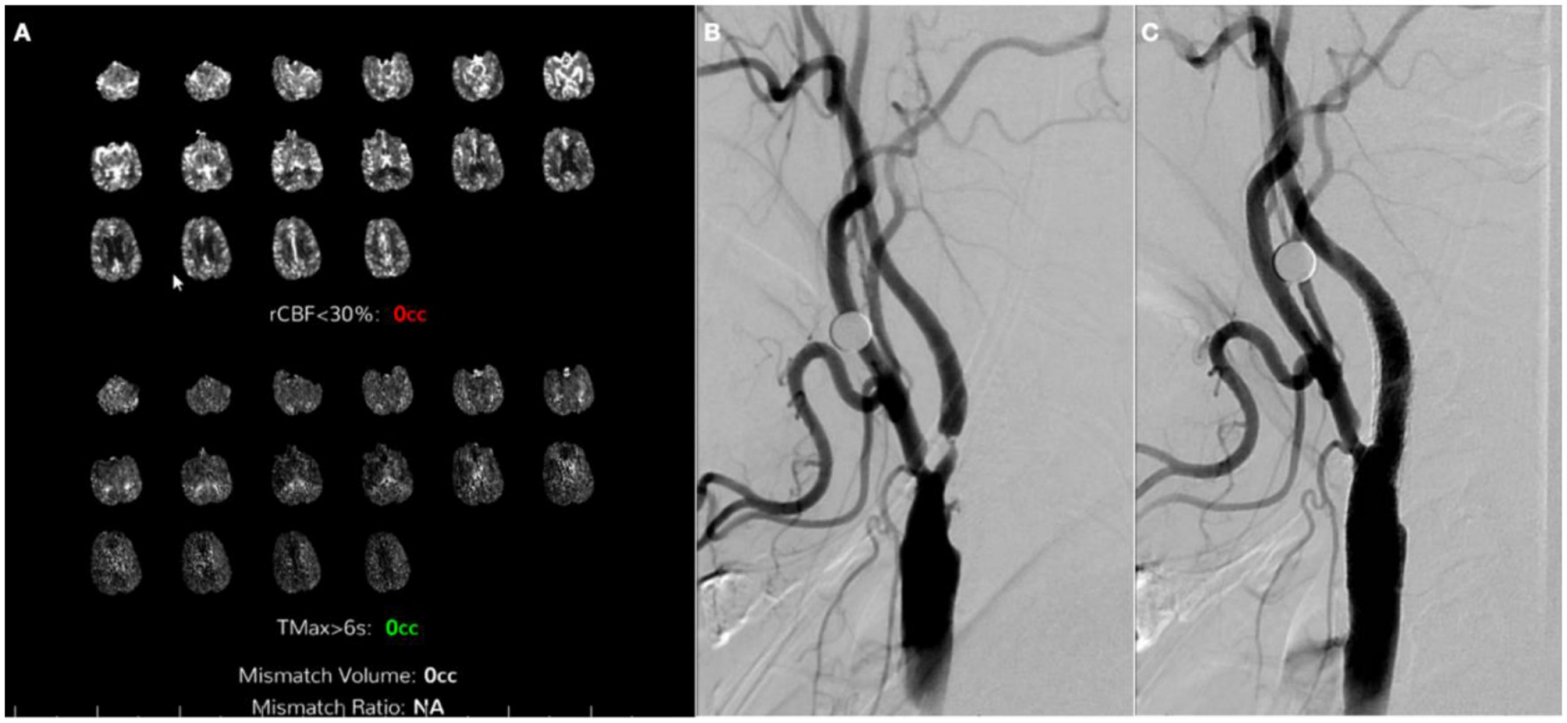
Computerized tomography perfusion (CTP) and digital subtraction angiography 1 month after contralateral stenting. **(A)** CTP 28 days after right internal carotid artery stenting showing no core or penumbra on left middle cerebral artery territory; **(B)** Left common carotid artery (CCA) injection, neck lateral view pre-stenting; **(C)** Left CCA injection, neck lateral view post-stenting.

Four weeks after the first procedure, the patient was readmitted for the second stage of stenting. There was interval clinical improvement with only residual minimal aphasia. The patient finally underwent left ICA stenting without complications (Figure 5). On a 90-day follow-up assessment, the patient continued to improve to full resolution of previous weakness and speech impairment and was able to resume their previous activities.

## Discussion

The management of bilateral severe carotid stenosis has not been separately addressed by the different guidelines on CAD management over the last two decades (Abbott et al., 2015; Bonati et al., 2021), although single-center experience reports have suggested different approaches may be feasible. We decided to report this case due to our unique approach involving intravenous thrombolytic use, fighting the temptation to pursue mechanical thrombectomy, and the delayed staged stenting of both carotid arteries, starting with the asymptomatic side to prevent the risk of hyperperfusion, based on clinical improvement and angiographic collateral flow.

Our patient presented in the morning of symptom recognition after last seen well the prior evening at bedtime. Despite the unclear timing of symptom onset, wake-up strokes have now been addressed by different randomized clinical trials (Davis et al., 2008; Ma et al., 2019; Ringleb et al., 2019; Thomalla et al., 2018). After the publication of the WAKE-UP trial, some of these trials lost clinical equipoise and had to be terminated before achieving their predetermined sample size. Nonetheless, a meta-analysis of four trials that used CT or MRI-based perfusion methods to indicate thrombolytic use showed benefits in functional outcomes (Campbell et al., 2019).

Despite the patient also planning for endovascular intervention for acute proximal left ICA occlusion (ICAO) with initially evolving deficits, the interval clinical improvement associated with recanalization of proximal ICA, as well as angiographic evidence of collateral supply to main left MCA territory through Acomm and leptomeningeal vessels, leading to the deferral of the procedure. In acute ICAO, CAS performance has been reported mostly in retrospective single-center studies, with excellent recanalization rates and worse outcomes, particularly in tandem occlusions (Jovin et al., 2005). Successful recanalization is known to be affected by factors such as length of the occlusion, composition of thrombus or plaque, and adequacy of collateral circulation (Tang et al., 2022).

Besides the technical difficulties, even successful recanalization of acute cervical ICAO may be associated with complications such as procedural hemodynamic depression, myocardial infarction (MI), and HPS following the procedure. HPS has been associated with symptomatic stenosis of >90%, contralateral stenosis ≥80%, and poor intracerebral collaterals (Abou-Chebl et al., 2004; Coutts et al., 2003). Our patient had all these risk factors except for a relatively preserved collateral flow, predominantly through Acomm, yet it was diminished due to the severe right carotid stenosis.

To date, there have been 15 single-center case series of patients undergoing simultaneous or staged bilateral carotid interventions in which authors accounted for hyperperfusion syndrome as a potential complication (Table 1). Most of these series evaluated only simultaneous bilateral CAS (SimBCAS), but four included both SimBCAS and staged bilateral carotid stenting (BCAS) with segregated outcomes (Henry et al., 2005; Li et al., 2014; Liu et al., 2010; Oshita et al., 2020). Across all series, HPS was only reported in one case of staged BCAS (Henry et al., 2005), while it affected up to 16% of cases of SimBCAS (Henry et al., 2005; Li et al., 2014; Liu et al., 2010; Oshita et al., 2020). There was no fatal outcome in the patients who underwent staged BCAS, but there were two deaths in patients who had SimBCAS, one of which was attributed to HPS (Henry et al., 2005).

**Table 1:** Complication rates in simultaneous and staged bilateral carotid interventions.

**Study authors, year**	**Patients undergoing bilateral intervention**	**Interventions by type (*n*)**	**HPS**	**Stroke**	**MI**	**Death**
	***n***		***n*** **(%)**	***n*** **(%)**	***n*** **(%)**	***n*** **(%)**
Alurkar et al. (2012)	9	SimBCAS (9)	0	0	0	0
Chen et al. (2004)	10	SimBCAS (10)	0	0	0	0
Dong et al. (2012)	39	SimBCAS (39)	1 (2.6%)	2 (5.1%)	1 (2.6%)	1 (2.6%)
Hashimoto et al. (2023)	3	Staged BCAS	0	0	0	0
Henry et al. (2005)	57	SimBCAS (17)	1 (5.8%)	1 (5.8%)	1 (5.8%)	2 (11.7%)
		Staged BCAS (40)	1 (2.5%)	0	0	0
Hokari et al. (2014)	2	Staged ipsilateral CEA, contraleral CAS	1 (50%)	0	0	0
Jiang et al. (2016)	120	SimBCAS (120)	3 (2.5%)	5 (4.2%)	1 (0.8%)	1 (0.8%)
Lee et al. (2006)	27	SimBCAS (21), staged BCAS (6)	0	1 (3.7%)	0	0
Li et al. (2014)	42	SimBCAS (42)	2 (4.8%)	1 (2.4%)	0	0
		Staged BCAS (26)	0	1 (3.8%)	0	0
Liu et al. (2010)	30	SimBCAS (24)	4 (16.7%)	2 (8.3%)	NR	1 (4.2%)
		Staged BCAS (6)	0	0	NR	0
Oshita et al. (2020)	12	SimBCAS (8)	0	0	0	0
		Staged BCAS (4)	0	0	0	0
Shchehlov et al. (2022)	39	SimBCAS (39)	2 (5.1%)	2 (5.1%)	0	0
Wang et al. (2008)	6	SimBCAS (6)	0	0	0	0
Xu et al. (2016)	8	Same-day ipsilateral CEA, contralateral CAS (5)	1 (20%)	0	0	0
		Same-day contralateral CAS, ipsilateral CEA (3)	0	0	0	0
Ye et al. (2016)	70	SimBCAS (70)	4 (5.7%)	1 (1.4%)	0	0

HPS, hyperperfusion syndrome; MI, myocardial infarction; SimBCAS, simultaneous bilateral carotid artery stenting; BCAS, bilateral carotid artery stenting; CEA, carotid endarterectomy; CAS, carotid artery stenting; NR, not reported.

Among patients who undergo bilateral staged interventions, several approaches have been reported by different centers (Diehm et al., 2008; Fukuda et al., 2003; Liu et al., 2015; Xu et al., 2016). In a series of eight patients with bilateral carotid stenosis who underwent same-day CEA of the symptomatic side and CAS of the asymptomatic side, the authors opted to start with CAS of the asymptomatic side in three patients that had ACA of the side planned to undergo CEA supplied by contralateral side, similarly to our approach in this case. The remaining five patients had CEA of the symptomatic side first. Although both groups did not suffer stroke, MI, or death, there was one case of hyperperfusion injury in the group that had CEA of the symptomatic carotid first (Xu et al., 2016).

We opted to pursue our first stage a few days later and the second stage 4 weeks after the first intervention. The timing of intervention in symptomatic carotid stenosis has been a topic of debate among interventionists, but a recent systematic review suggests better outcomes when it is pursued within 2 weeks of first symptoms (Rerkasem et al., 2020). There has been no consensus, however, on the optimal timing of the second procedure in staged BCAS. Five case series reported the timing of staged CAS in a total of 75 patients. Except for one of these studies, in which patients had a staged intervention done 24 h to 1 week after the first procedure (Henry et al., 2005), all the other studies reported second-stage CAS 1–12 weeks after the first intervention (Hashimoto et al., 2023; Henry et al., 2005; Hokari et al., 2014; Li et al., 2014; Oshita et al., 2020).

Although this has not been the first case of staged stenting starting from the asymptomatic side (Hashimoto et al., 2023; Xu et al., 2016), it is the first case to the authors' knowledge in which a patient preemptively received IVT in extended window before planned staged BCAS with interval improvement in ipsilateral anterograde flow, as well as improvement in perfusion imaging suggestive of optimized collateral flow and cerebrovascular autoregulation.

Our approach may not be extrapolated to every case of bilateral severe carotid stenosis, but it highlights the importance of staged stenting in bilateral carotid stenosis due to the higher risk of HPS. In selected cases, this could be attenuated by CAS of the asymptomatic carotid first, followed by delayed stenting of the symptomatic carotid after optimization of collateral flow based on angiographic and perfusion imaging.

## Conclusion

Carotid artery stenosis is a major risk factor for stroke. Bilateral carotid artery stenosis poses a challenge to interventionists in deciding the optimal approach, sequence, and timing of bilateral procedures due to the additional risk of hyperperfusion syndrome even after uncomplicated recanalization. In these patients, a tailored approach based on the patient's symptomatic side and angiographic and perfusion imaging features may provide the safest approach to avoid hyperperfusion, often starting with the intervention of the asymptomatic carotid side.

## Data Availability

The original contributions presented in the study are included in the article/supplementary material, further inquiries can be directed to the corresponding author.

## References

[B1] Abbott A. L. Paraskevas K. I. Kakkos S. K. Golledge J. Eckstein H. H. Diaz-Sandoval L. J.. (2015). Systematic review of guidelines for the management of asymptomatic and symptomatic carotid stenosis. Stroke 46, 3288–3301. 10.1161/STROKEAHA.115.003390 26451020

[B2] Abou-Chebl A. Yadav J. S. Reginelli J. P. Bajzer C. Bhatt D. Krieger D. W. (2004). Intracranial hemorrhage and hyperperfusion syndrome following carotid artery stenting: risk factors, prevention, and treatment. J. Am. Coll. Cardiol. 43, 1596–1601. 10.1016/j.jacc.2003.12.039 15120817

[B3] Alurkar A. Karanam L. S. P. Nayak S. Oak S. (2012). Simultaneous bilateral carotid stenting in a series of 9 patients: a single-center experience with review of literature. J. Clin. Imaging Sci. 2:72. 10.4103/2156-7514.104305 23393629 PMC3551520

[B4] Bonati L. H. Kakkos S. Berkefeld J. de Borst G. J. Bulbulia R. Halliday A.. (2021). European Stroke Organisation guideline on endarterectomy and stenting for carotid artery stenosis. Eur. Stroke J. 6, I–XLVII. 10.1177/23969873211012121 34414302 PMC8370069

[B5] Campbell B. C. V. Ma H. Ringleb P. A. Parsons M. W. Churilov L. Bendszus M.. (2019). Extending thrombolysis to 4·5–9 h and wake-up stroke using perfusion imaging: a systematic review and meta-analysis of individual patient data. Lancet 394, 139–147. 10.1016/S0140-6736(19)31053-0 31128925

[B6] Chaturvedi S. Bruno,; A., Feasby,; T., Holloway,; R., Benavente,; O., Cohen,; S. N.,. (2005). Carotid endarterectomy-an evidence-based review report of the therapeutics and technology assessment subcommittee of the American Academy of Neurology. Am. J. Opthalmol. 41, 238–239. 10.1016/j.ajo.2005.11.031 16186516

[B7] Chen M. S. Bhatt D. L. Mukherjee D. Chan A. W. Roffi M. Kapadia S. R.. (2004). Feasibility of simultaneous bilateral carotid artery stenting. Catheteriz. Cardiovasc. Interv. 61, 437–442. 10.1002/ccd.10742 15065133

[B8] Coutts S. B. Hill M. D. Hu W. Y. Sutherland G. R. Findlay J. M. Dempsey R. J.. (2003). Hyperperfusion syndrome: toward a stricter definition. Neurosurgery. 53, 1053–1058. 10.1227/01.NEU.0000088738.80838.74 14580271

[B9] Davis S. M. Rey G. Donnan A. Parsons M. W. Levi C. Butcher K. S.. (2008). Articles effects of alteplase beyond 3 h after stroke in the Echoplanar Imaging Thrombolytic Evaluation Trial (EPITHET): a placebo-controlled randomised trial. Lancet Neurol. 7, 299–309. 10.1016/S1474-4422(08)70044-9 18296121

[B10] Diehm N. Katzen B. T. Iyer S. S. White C. J. Hopkins L. N. Kelley L. (2008). Staged bilateral carotid stenting, an effective strategy in high-risk patients - insights from a prospective multicenter trial. J. Vasc. Surg. 47, 1227–1234. 10.1016/j.jvs.2008.01.035 18440179

[B11] Dong H. Jiang X. J. Peng M. Ji W. Wu H. Y. Hui R. T.. (2012). Comparison of the safety of simultaneous bilateral carotid artery stenting versus unilateral carotid artery stenting: 30-day and 6-month results. Chinese Med. J. 125, 1010–1015. 10.3760/cma.j.issn.0366-6999.2012.06.010 22613523

[B12] Fukuda H. Iihara K. Sakai N. Murao K. Sakai H. Higashi T.. (2003). staged carotid stenting and carotid endarterectomy for bilateral internal carotid artery stenosis. Intervent. Neuroradiol. 9, 143–148. 10.1177/15910199030090S120 20591244 PMC3553470

[B13] Hashimoto K. Yoshioka H. Kanemaru K. Senbokuya N. Kinouchi H. (2023). A novel staged revascularization strategy for bilateral severe internal carotid artery stenosis at high risk for hyperperfusion syndrome. World Neurosurg. 177, e294–e299. 10.1016/j.wneu.2023.06.036 37331474

[B14] Henry M. Gopalakrishnan L. Rajagopal S. Rath P. C. Henry I. Hugel M. (2005). Bilateral carotid angioplasty and stenting. Catheteriz. Cardiovasc.Interv. 64, 275–282. 10.1002/ccd.20287 15736256

[B15] Hokari M. Isobe M. Asano T. Itou Y. Yamazaki K. Chiba Y.. (2014). Treatment strategy for bilateral carotid stenosis: 2 cases of carotid endarterectomy for the symptomatic side followed by carotid stenting. J. Stroke Cerebrovasc. Dis. 23, 2851–2856. 10.1016/j.jstrokecerebrovasdis.2014.07.014 25280820

[B16] Jiang X. J. Dong H. Peng M. Zou Y. B. Song L. Xu B.. (2016). Simultaneous bilateral vs unilateral carotid artery stenting: 30-day and 1-year results. J. Endovasc. Ther. 23, 258–266. 10.1177/1526602815626900 26823486

[B17] Jovin T. G. Gupta R. Uchino K. Jungreis C. A. Wechsler L. R. Hammer M. D.. (2005). Emergent stenting of extracranial internal carotid artery occlusion in acute stroke has a high revascularization rate. Stroke 36, 24262430. 10.1161/01.STR.0000185924.22918.51 16224082

[B18] Lee Y. Kim T. Suh S. Kwon B. Lee T. Kwon O.. (2006). Simultaneous bilateral carotid stenting under the circumstance of neuroprotection device. Interv. Neuroradiol. 12, 141–148. 10.1177/159101990601200208 20569566 PMC3354519

[B19] Li Y. Sun W.en Sun W.enshan Yin Q. Wang Y. Cai Q. Huang Z. Liu W.. (2014). Safety and efficacy of simultaneous bilateral carotid angioplasty and stenting. J. Thromb. Thrombolysis 37, 202–209. 10.1007/s11239-013-0920-1 23553247

[B20] Liu B. Wei W. Wang Y. Yang X. Yue S. Zhang J. (2015). Treatment strategy for bilateral severe carotid artery stenosis: one center's experience. World Neurosurg. 84, 820–825. 10.1016/j.wneu.2015.03.067 25871783

[B21] Liu S. Jung J. H. Kim S. M. Lim H. K. Kwon H. J. Kim J. K.. (2010). Simultaneous bilateral carotid stenting in high-risk patients. Am. J. Neuroradiol. 31, 1113–1117. 10.3174/ajnr.A1970 20053810 PMC7963934

[B22] Ma H. Campbell B. C. V. Parsons M. W. Churilov L. Levi C. R. Hsu C.. (2019). Thrombolysis guided by perfusion imaging up to 9 hours after onset of stroke. N. Engl. J. Med. 380, 1795–1803. 10.1056/NEJMoa1813046 31067369

[B23] Moulakakis K. G. Mylonas S. N. Sfyroeras G. S. Andrikopoulos V. (2009). Hyperperfusion syndrome after carotid revascularization. J. Vasc. Surg. 49, 10601068. 10.1016/j.jvs.2008.11.026 19249185

[B24] Oshita J. Sakamoto S. Okazaki T. Ishii D. Kurisu K. (2020). Safety of simultaneous bilateral carotid artery stenting for bilateral carotid artery stenosis. Interv. Neuroradiol. 26, 19–25. 10.1177/1591019919869478 31423862 PMC6997998

[B25] Ouriel K. Shorteu C. K. Illig K. A. Greenberg R. I. Green R. M. (1999). Intracerebral hemorrhage after carotid endarterectomy: incidence, contribution to neurologic morbidity, and predictive factors. J. Vasc. Surg. 29, 82–89. 10.1016/S0741-5214(99)70362-9 9882792

[B26] Rerkasem A. Orrapin S. Howard D. P. J. Rerkasem K. (2020). Carotid endarterectomy for symptomatic carotid stenosis. Cochrane Database Syst. Rev. 9:CD001081. 10.1002/14651858.CD001081.pub4 32918282 PMC8536099

[B27] Ringleb P. Bendszus M. Bluhmki E. Donnan G. Eschenfelder C. Fatar M.. (2019). Extending the time window for intravenous thrombolysis in acute ischemic stroke using magnetic resonance imaging-based patient selection. Int. J. Stroke 14, 483–490. 10.1177/1747493019840938 30947642

[B28] Shchehlov D. V. Svyrydiuk O. Y. Vyval M. B. Sydorenko O. F. Nosenko N. M. Gudym M. S. (2022). Simultaneous bilateral angioplasty and stenting for carotid stenosis - a single center experience. J. Med. Life 15, 252–257. 10.25122/jml-2021-0274 35419100 PMC8999088

[B29] Son S. Choi D. S. Kim S. K. Kang H. Park K. J. Choi N. C.. (2013). Carotid artery stenting in patients with near occlusion: a single-center experience and comparison with recent studies. Clin. Neurol. Neurosurg. 115, 1976–1981. 10.1016/j.clineuro.2013.06.001 23820331

[B30] Song P. Fang Z. Wang H. Cai Y. Rahimi K. Zhu Y.. (2020). Global and regional prevalence, burden, and risk factors for carotid atherosclerosis: a systematic review, meta-analysis, and modelling study. Lancet Glob. Health 8, e721–e729. 10.1016/S2214-109X(20)30117-0 32353319

[B31] Tang M. Yan X. Gao J. Li L. Zhe X. Zhang X.. (2022). High-resolution MRI for evaluation of the possibility of successful recanalization in symptomatic chronic ICA occlusion: a retrospective Study. Am. J. Neuroradiol. 43, 1164–1171. 10.3174/ajnr.A7576 35863780 PMC9575431

[B32] Thomalla G. Simonsen C. Z. Boutitie F. Andersen G. Berthezene Y. Cheng B.. (2018). MRI-guided thrombolysis for stroke with unknown time of onset. N. Engl. J. Med. 379, 611–622. 10.1056/NEJMoa1804355 29766770

[B33] Wang Y. H. Hsieh H. J. Lee C. W. Chen Y. F. Jeng J. S. Liu H. M. (2008). Simultaneous bilateral carotid stenting in one session in high-risk patients. J. Neuroimaging 18, 252–255. 10.1111/j.1552-6569.2007.00208.x 18304035

[B34] Xu R. wei Liu P. Fan X. qiang Wang Q. Zhang J. bin Ye Z. dong (2016). Feasibility and safety of simultaneous carotid endarterectomy and carotid stenting for bilateral carotid stenosis: a single-center experience using a hybrid procedure. Ann. Vasc. Surg. 33, 138–143. 10.1016/j.avsg.2015.11.017 26902940

[B35] Ye Z. Liu Y. Deng X. Chen X. Lin C. Tang Y.. (2016). Simultaneous bilateral carotid stenting for symptomatic bilateral high-grade carotid stenosis: a retrospective clinical investigation. Med. Sci. Monit. 22, 2924–2933. 10.12659/MSM.896505 27542158 PMC4994931

